# Evaluation of lung cancer early detection offered by the German Social Accident Insurance for formerly asbestos-exposed employees using low-dose computed tomography – setting and study design

**DOI:** 10.1186/s13690-025-01662-9

**Published:** 2025-07-30

**Authors:** Felix Greiner, Jan Heidrich, Helena Keller, Dirk Taeger, Thorsten Wiethege, Volker Harth

**Affiliations:** 1 Institute for Occupational and Maritime Medicine (ZfAM), University Medical Center Hamburg-Eppendorf (UKE), Seewartenstraße 10, 20459 Hamburg, Germany; 2 Institute for Prevention and Occupational Medicine of the German Social Accident Insurance, Institute of the Ruhr University Bochum (IPA), Bochum, Germany

**Keywords:** Lung cancer screening, Early detection, Low-dose computed tomography (LDCT), Occupational disease, Asbestos dust exposure, Decision aid, Perception, Quality indicators

## Abstract

**Background:**

Clinical trials have shown the benefits of lung cancer screening (LCS) in certain high-risk groups using low-dose high-resolution computed tomography (LDCT). Risk groups are usually defined by age and tobacco use. Exposure to asbestos dust is an important occupational risk factor for lung cancer. Since 2014, the German Social Accident Insurance (DGUV) has introduced annual LCS for high-risk groups (EVA-LCS). In addition to occupational asbestos dust exposure, the population at risk is defined by age (≥ 55 years) and tobacco consumption (≥ 30 pack-years). The health services research project EVALUNG aims to evaluate the EVA-LCS using a combination of quantitative and qualitative methods.

**Methods:**

The quantitative part will be based on a secondary data analysis of routine administrative and medical data from the EVA-LCS. The results of the individual screening rounds will be analysed in a cross-sectional design. Primary endpoints are participation patterns, the rate of findings requiring further diagnostic investigation, the detection of lung cancer including tumour stage and characteristics, and the notification and recognition of asbestos-related occupational diseases. Secondary endpoints include false-positive and false-negative findings, incidence of other cancers, and all-cause and cancer-related mortality. To avoid selection bias, a complete set of anonymised data (approximately 22,200 individuals as of 12/2021) from the EVA-LCS will be transmitted for use in EVALUNG. A sub-sample will be used to perform longitudinal analyses and explore a linkage with cancer registry data. Another component is the development and piloting of quality indicators. Qualitative interviews will be conducted to analyse the perceptions, satisfaction, and potential psychological effects of EVA-LCS participants. Interviews with participating physicians will focus on their attitudes and knowledge regarding LCS. A further aim is to develop an evidence-based decision aid.

**Discussion:**

The EVALUNG concept is based on various complementary approaches, enabling a comprehensive evaluation of the EVA-LCS and the identification of optimization potentials. The quality of the data is crucial for the validity of the quantitative analyses. One way to address potential limitations is to link the data with cancer registry data. The results may contribute to the planning and development of a national LDCT lung cancer screening programme in Germany.

**Table:** 

**Text box 1. Contributions to the literature**	
• The German Social Accident Insurance offers lung cancer screening for high-risk groups, defined by a history of occupational asbestos dust exposure, age and tobacco consumption.	
• This study protocol was designed to evaluate the processes and outcomes of the screening. It addresses the conduct of qualitative interviews with participants and physicians, and the challenges inherent to the use of routine data for quantitative analyses.	
• The study has the advantage that data from non-participants can be employed for comparative analyses.	
• The findings of the evaluation may provide guidance for the implementation of a population-based lung cancer screening programme in Germany.	

## Background

Of all new cancer cases in Germany each year, lung cancer is the second most common in men and the third most common in women. Furthermore, lung cancer is by far the most common fatal cancer in men and second most in women, accounting for about 22% (men) and 16% (women) of tumour-related deaths in Germany in 2020 [1]. This is because early-stage lung cancer rarely causes clinical symptoms and is therefore usually detected at late-stage, while curative treatment tends to be successful only at an early stage.

Because of the social importance of lung cancer, several randomised controlled trials (RCTs) of lung cancer screening (LCS) using low-dose high-resolution computed tomography (LDCT) have been conducted in recent years. Most prominent are the two studies with the largest sample sizes; the National Lung Screening Trial (NLST) [2] from the USA and the Nederlands-Leuvens Longkanker Screenings Onderzoek (NELSON) study from Europe [3]. Meta-analyses of these and other randomized controlled trials (RCTs) demonstrate that LCS with LDCT reduces disease-specific mortality in risk groups of older current or former smokers by 12–21%, depending on the duration of follow-up [4–6].

The RCTs on LDCT screening refer to high-risk groups for lung cancer, defined mainly by the criteria of tobacco smoking and age [7], but not by other factors, in particular occupational asbestos dust exposure (OAE). The carcinogenicity of asbestos dust has long been recognised and proven beyond doubt [8]. It is estimated that 4.5–6.9% of all lung cancer cases in Germany are associated with OAE [9]. It is assumed that there is a synergistic effect for the interaction between OAE and tobacco smoking, which is relevant due to the frequent co-occurrence of OAE and tobacco use [10]. For ethical reasons, it is not possible to conduct RCTs and generate direct evidence from RCTs on the benefits of LDCT screening in individuals with OAE. Instead, evidence is derived indirectly from two meta-analyses that summarised existing studies on the benefits of LDCT screening in the context of occupational asbestos exposure [11, 12]. The pooled detection rates of 1.1% and 0.8%, respectively, are similar to those found in the first screening round of RCTs in heavy smokers. The high proportion (57%) of tumours detected in stage I in one study, which have a more favourable prognosis, was also comparable [13]. It is concluded that the reduction in mortality seen in the RCTs with smokers can be extrapolated to people with OAE [11].

An observational study evaluated a comprehensive surveillance programme in Italy [14]. Lung cancer mortality was shown to be reduced by LDCT screening in men with OAE compared with regular follow-up with physical examination and chest X-ray. This study confirms the results of a previous study conducted on 576 German individuals with high exposure to asbestos dust [15]. The participants underwent annual LDCT between 1993 and 1997 and were followed up until 2007. A standardised mortality ratio (SMR) for lung cancer mortality of 0.39 (confidence interval 0.17–0.77) was found, corresponding to a mortality reduction in the range of 23–83%.

Based on the results of the NLST study, international medical-scientific societies had recommended LCS with LDCT for populations with a corresponding risk constellation (overviews e.g. in Sands et al. [16] and Silva et al. [17]). The recommendations for inclusion criteria vary and consider factors such as age, smoking history, other risk factors, and model-based lung cancer risk.

The National Comprehensive Cancer Network (NCCN) guideline from the USA is the only one to date that explicitly includes OAE as an inclusion criterion. Regarding asbestos, the NCCN recommendations are not based on RCTs, but on findings from observational studies. LDCT screening is recommended for individuals with OAE and a smoking history of at least 20 pack-years from the age of 50, regardless of the duration of smoking cessation [18].

Based on the scientific evidence and recommendations of the above-mentioned medical guidelines, the German Social Accident Insurance^1^ (Deutsche Gesetzliche Unfallversicherung – DGUV) offers a preventive occupational healthcare programme for the early detection of lung cancer (Erweitertes Vorsorgeangebot der DGUV zur Früherkennung von Lungenkrebs – EVA-Lunge). It is called “extended preventive healthcare” as it is an additional component for the mandatory follow-up preventive health care for employees with a history of OAE. Following its launch in pilot regions from 2014, that programme (further referred to as EVA-LCS) has been introduced nationwide in Germany since 2017. In addition to OAE, the risk population is also defined by age and tobacco smoking.

Guidelines and medical associations recommend that LCS should only be performed under standardised conditions, accompanied by quality management and systematic evaluation [19, 20].

The health services research project EVALUNG was designed to evaluate the processes and outcomes of the EVA-LCS, using a combination of quantitative and qualitative methods. The quantitative evaluation is based on routine administrative and medical data from the EVA-LCS. Therefore, we will first describe the EVA-LCS in detail before presenting the EVALUNG study protocol.

## Setting: Lung cancer screening offered by the German Social Accident Insurance

### Inclusion criteria

The EVA-LCS itself is not a scientific study but a regular preventive occupational healthcare programme. It is available for two cohorts, which differ in the definition of the occupational asbestos exposure (Table 1). Further eligibility criteria for both cohorts are age ≥ 55 years and a smoking history of ≥ 30 pack years. Participation in the EVA-LCS is free of charge and voluntary [21].

The main cohort (so-called GVS cohort) is defined by an OAE of ≥ 10 years with onset before 1985. The GVS cohort is administered by the “Preventive Healthcare Service” (Gesundheitsvorsorge – GVS), a joint organisation of the individual German social accident insurance institutions (Unfallversicherungsträger - UVT) within the DGUV.

The GVS organises follow-up preventive health care for employees who were exposed to silicogenic, asbestos or artificial mineral dusts during their occupational history. The GVS follow-up care begins after employment with occupational (asbestos) dust exposure has ended and ensures that occupational health care continues beyond the employee’s working life. In principle, the employer is obliged to provide medical follow-up. However, at the end of an employment relationship this obligation can be, and usually is, transferred to the relevant accident insurance institution, which entrusts the GVS with the coordination.

The second cohort (UVT cohort) includes insured persons, who have already been diagnosed with asbestosis, which is recognized as an occupational disease (OD) no. 4103 (asbestosis or diseases of the pleura caused by asbestos dust) according to the Ordinance on Occupational Diseases, Annex 1. The UVT cohort is managed by one of the participating UVT^2^.

The legal basis for the EVA-LCS is the Ordinance on Preventive Occupational Health Care (ArbMedVV), paragraph 5 (3), sentence 2, in the case of the GVS cohort. For the UVT cohort, it is the German Social Code Book (SGB) VII, paragraph 26 (2) no. 1. The objective of the GVS cohort is to provide follow-up preventive health care, whereas the objective of the UVT cohort is to prevent the deterioration of an existing OD, also a responsibility of statutory accident insurance.

The LDCT scans are performed in accordance with the Radiation Protection Act (StrlSchG), paragraph 83 (1) no. 2 as so-called “non-medical applications” within the provisions of general occupational health and safety. They do not constitute early detection in the sense of StrlSchG, paragraph 5 (16). Therefore, they do not require an ordinance in accordance with StrlSchG, paragraph 84 (2), as is normally required for early detection measures using ionizing radiation [22].

There are no general exclusion criteria for EVA-LCS. Individual exclusion criteria may be determined during the mandatory medical consultation (see below).

**Table 1:** Description of the two cohorts eligible for lung cancer screening offered by the German Social Accident Insurance since 2014

	GVS cohort	UVT cohort
**Responsible institution**	Preventive Healthcare Service (Gesundheitsvorsorge – GVS)	German Social Accident Insurance Institution (Berufsgenossenschaft – BG) for the building trade (BG BAU), BG for the energy, textile, electrical and media products sectors (BG ETEM), BG for the woodworking and metalworking industries (BGHM), BG for the trade and logistics industry (BGHW), BG for the raw materials and chemical industry (BG RCI), BG for the administrative sector (VBG)
**Inclusion criteria**		
- occupational asbestos dust exposure	≥ 10 years with onset before 1985	Recognized occupational disease (OD) no. 4103 (asbestosis or diseases of the pleura caused by asbestos dust)
- age	≥ 55 years	
- smoking history	≥ 30 pack years	
**Eligible persons** (as of 12/2021)	20,900	1,300

### Procedures within the EVA-LCS

Eligible persons are centrally identified either by the GVS or the respective UVT and invited to participate in EVA-LCS on an annual basis. They will receive a letter of invitation with basic information about the EVA-LCS and a consent form for referral to a medical consultation.

Medical counselling prior to LDCT scan is a mandatory component of EVA-LCS and supports shared decision-making [21]. It is provided by specialised physicians with a qualification in occupational medicine, lung specialists or pulmonologists. The medical consultation consists of a medical history, physical examination and counselling on LDCT. This includes the individual benefits of annual LDCT, but also associated risks such as the amount of radiation exposure. In addition, individual exclusion criteria are determined, e.g. a chest CT performed within the last 12 months or medical contraindications. In case of current smokers, the physician should also emphasise the risks associated with smoking and refer to smoking cessation programmes during the consultation. As those are not offered within the framework of EVA-LCS, interested persons are either given information material or are referred to their general practitioner.

If participants consent to have a LDCT scan, they will be referred to a collaborating radiology facility. The LDCT scans themselves follow a standardised protocol that includes an independent double assessment of abnormal findings as well as a random sample of all scans [23].

Depending on the LDCT finding, the following pathways are available for the further medical course within EVA-LCS:

- No suspicion of lung cancer, then invitation to participate again in 12 months.
- Suspicious finding with shortened follow-up interval according to NCCN guidelines [18].
- In case of suspected lung cancer further diagnostics, preferably in a certified lung cancer centre, and initiation of therapy, if necessary.
- Diagnosis or suspicion of further (incidental) findings with individual further procedure (e.g. in case of coronary calcification).

**Fig. 1:**
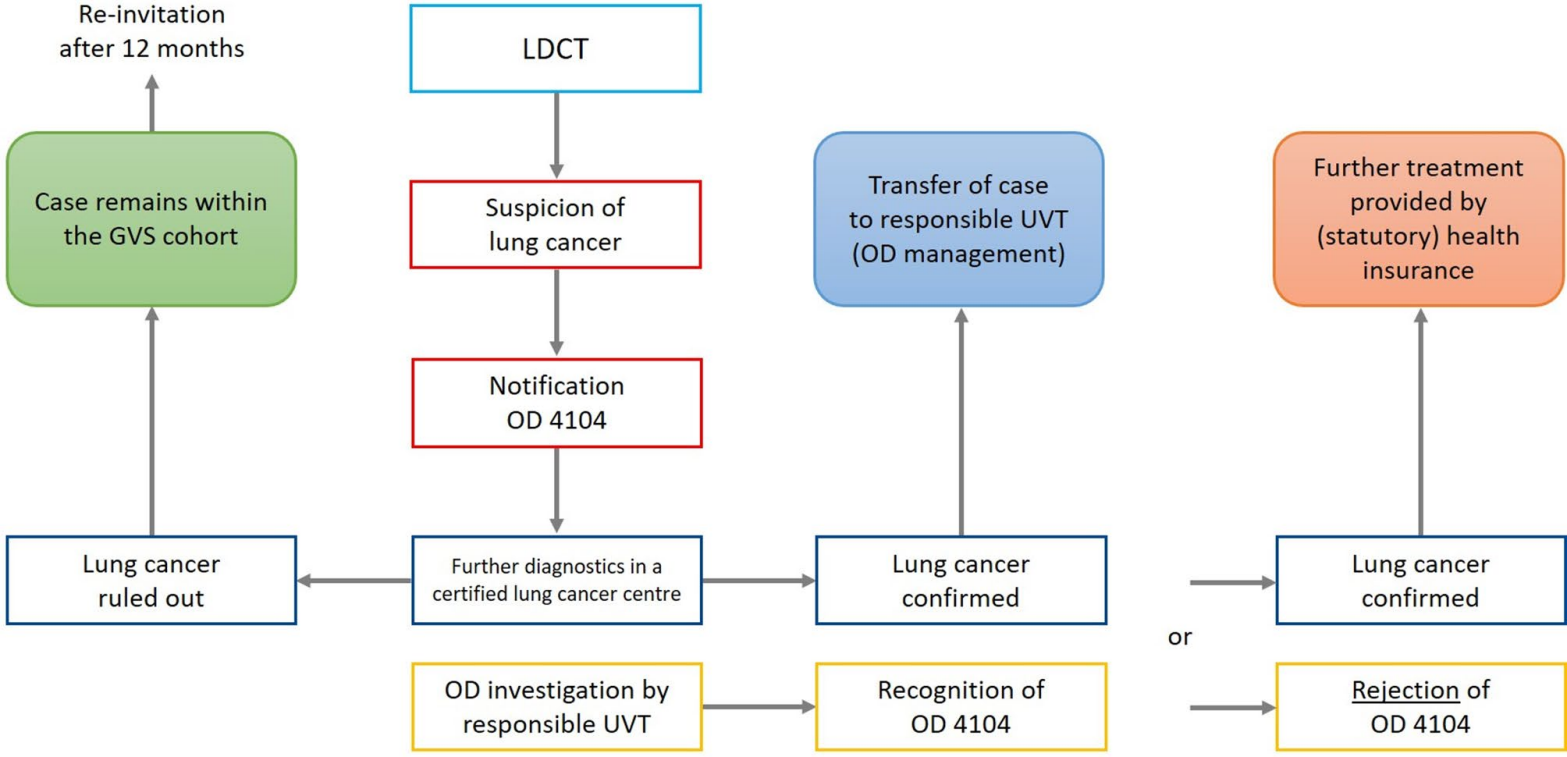
**a**: Flowchart of procedures within lung cancer screening offered by the German Social Accident Insurance (GVS cohort only) following a low-dose CT-scan that is suspicious for lung cancer (GVS Preventive Healthcare Service, OD 4104 occupational disease no. 4104 “lung cancer”, UVT Accident Insurance Institution)

In addition to the medical pathways, there are administrative aspects that need to be considered in the context of the occupational disease procedure of the statutory accident insurance scheme. In the case of suspected lung cancer, a notification of a suspected occupational disease (OD) no. 4104 (Lung, larynx or ovarian cancer caused by exposure to asbestos dust^3^) has to be registered. This is followed by a procedure in which the suspected OD is investigated by the responsible UVT (Fig. 1a). If the diagnosis of lung cancer is confirmed *and* sufficient occupational asbestos exposure is proven, *and* all other requirements of accident insurance law are met, the disease can be recognized as an OD no. 4104 of Annex 1 to the Ordinance on Occupational Diseases. In the case of recognised OD, the UVT covers the cost of treatment. In addition, recognition of OD is associated with the possibility of certain benefits, such as the payment of an OD pension in the event of occupational disability. In the event of rejection as an OD, the statutory health insurance (SHI) is obliged to cover the costs of treatment.

**Fig. 2:**
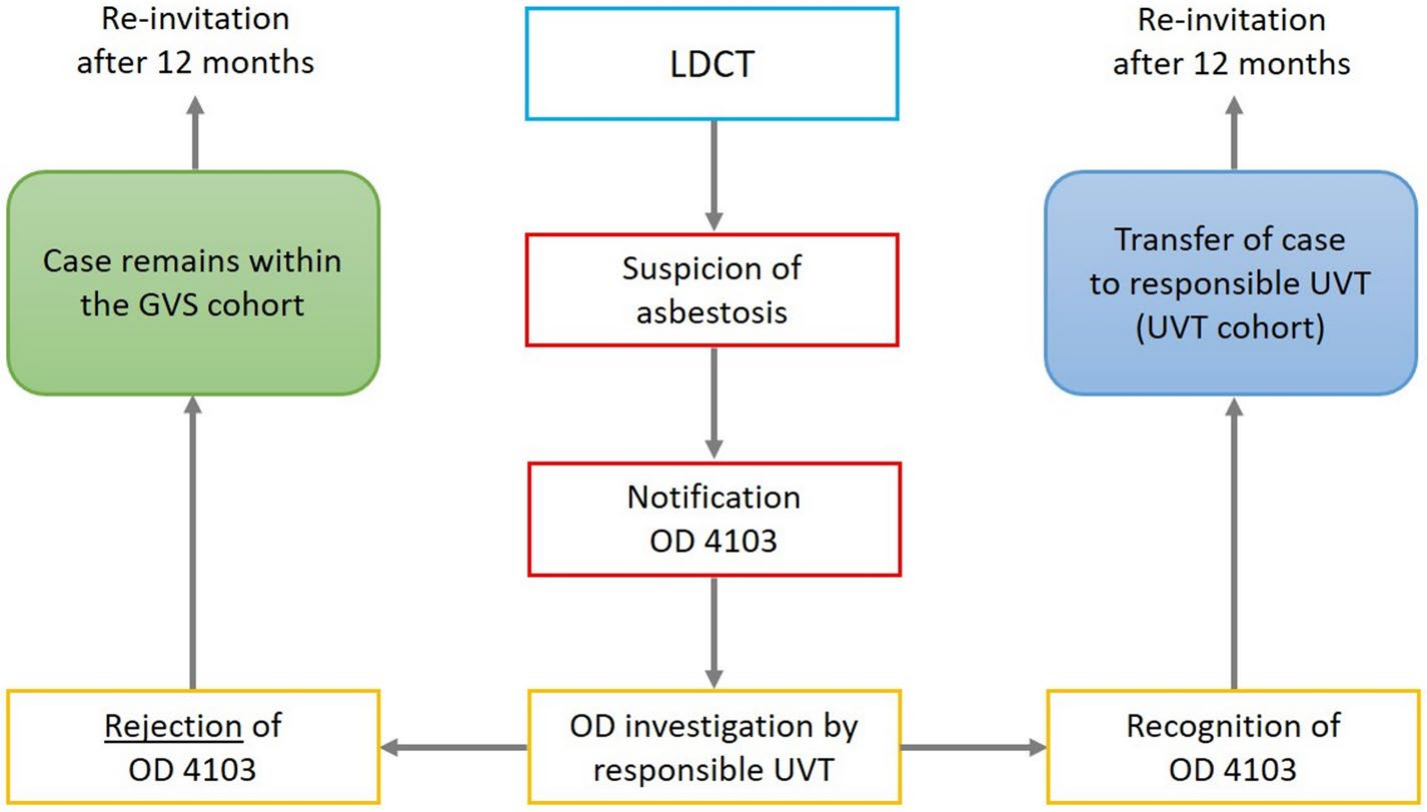
**b**: Flowchart of procedures within lung cancer screening offered by the German Social Accident Insurance (GVS cohort only) following a low-dose CT-scan that is suspicious for asbestosis (GVS Preventive Healthcare Service, OD 4103 occupational disease no. 4103 “asbestosis”, UVT Accident Insurance Institution)

It is also possible for a participant to be newly diagnosed with an asbestosis during EVA-LCS, which may be recognised as OD no. 4103 (Asbestosis or diseases of the pleura caused by asbestos dust). In these cases, the persons concerned will change from the GVS cohort to the UVT cohort and will be invited to the EVA-LCS by their respective UVT in the next screening round (Fig. 2b).

### Data collection within the EVA-LCS

The primary data holders are either the GVS, which acts on behalf of the UVT, or the UVT themselves. These institutions are mainly responsible for the coordination of the EVA-LCS. Further data sources include cooperating providers, such as the counselling physicians and radiological institutions.

**Fig. 3:**
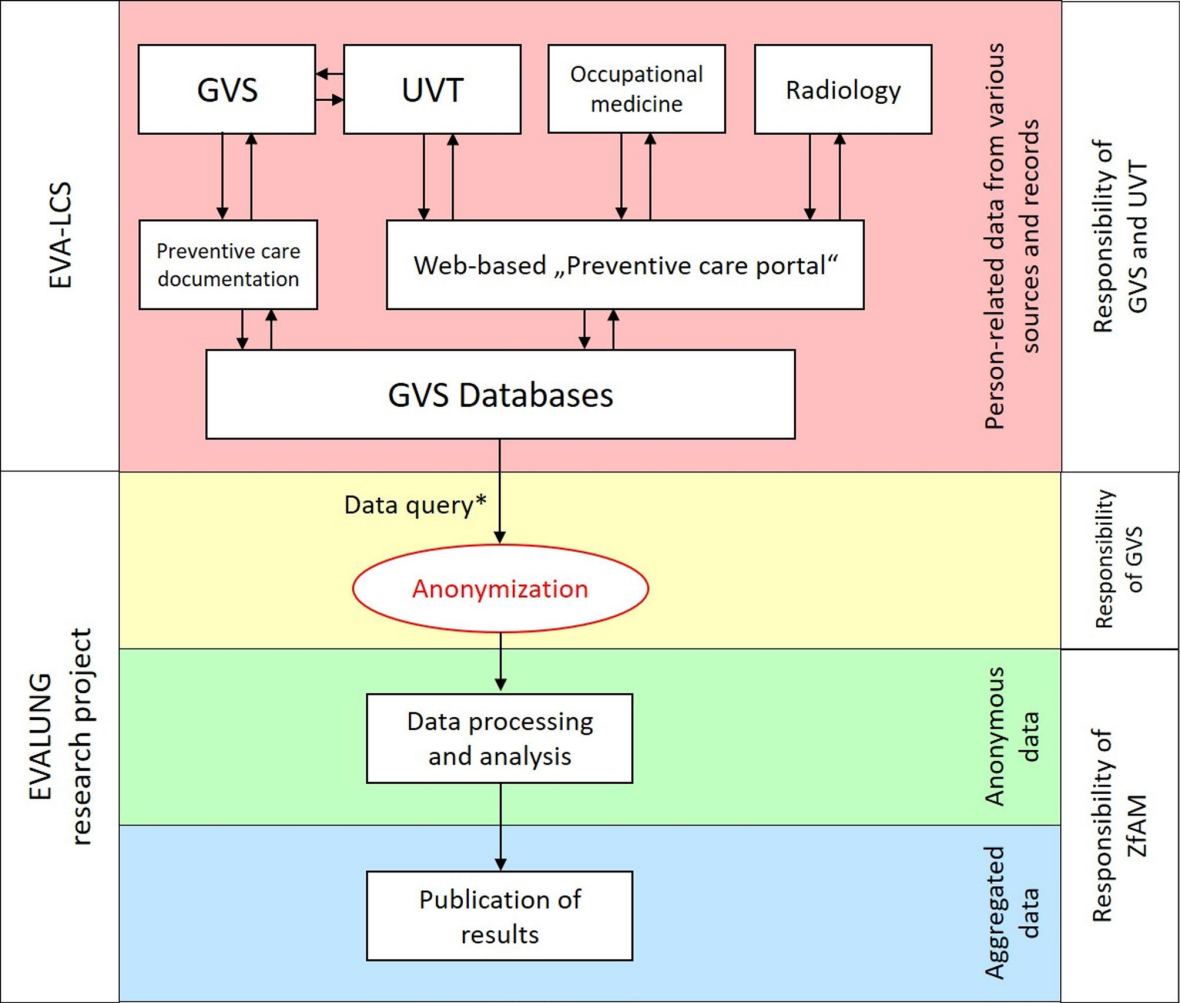
Flowchart of data management within lung cancer screening offered by the German Social Accident Insurance (EVA-LCS), including processing of data to be used within the EVALUNG research project (GVS Preventive Healthcare Service, UVT Accident Insurance Institutions, ZfAM Institute for Occupational and Maritime Medicine, * data extraction of selected variables according to the study protocol)

The standardised “Preventive care documentation” is managed under the responsibility of the GVS. A central web-based documentation platform (“Preventive care portal”) is available to participating UVT and external service providers for the direct input and transmission of, for example, appointments and medical results [24]. The data is stored in databases under the responsibility of the GVS (Fig. 3). In principle, data should always be entered directly and in a standardised way into the documentation platform. However, particularly in older cases, it may be that only a scanned document is stored in the central databases.

If there is no response to the invitation letter and the subsequent reminder, the GVS carries out regular checks on the current residential address and vital status via the residents’ registration offices. In case of death, the death certificate is requested to complete the “Preventive care documentation”, in particular the cause of death.

Routine documentation in the EVA-LCS currently consists of the following components:

1. Personal data and employment history data.
2. “Preventive care documentation” of the GVS and participating UVT containing selected administrative and medical content from the following records:
  Declaration of consent.
  Medical consultation record.
  LDCT record.
  Supplementary record in case of lung cancer (e.g. tumour classification).
3. Medical consultation record.
4. Classification of LDCT findings according to NCCN guidelines.
5. CT classification (ICOERD) from initial and secondary assessment.
6. Solid nodule record from initial and secondary assessment.
7. LDCT quality assurance record.
8. Supplementary record in case of lung cancer (e.g. tumour classification).

### Radiological quality management within the EVA-LCS

A concept for radiological quality management in the EVA-LCS has already been established. In summary, it consists of the following components [23].

- Continuous and automated quality control of all LDCT scans with regard to radiation dose,
- Double assessment of the first 10 scans from a newly participating radiological facility,
- Double assessment of all suspicious scans requiring a shortened follow-up or further (invasive) diagnostics,
- Double assessment of a random sample of all scans,
- Regular exchange of experience among the participating radiological facilities.

A comparison of the technical settings of the LDCT scans with the recommendations of the “Working Group for the Use of Diagnostic Radiology in Work-related and Environmental Diseases” (AG DRauE) [25] is carried out automatically in the “Preventive care portal”. Deviations from the standard protocol are immediately displayed upon data entry, and responsible radiologists and the GVS are automatically notified via email. Possible deviations are categorised using a traffic light system. If there are significant deviations (indicated by a red traffic light), the radiological facility will be prevented from participating in EVA-LCS until the issues have been reviewed and, if necessary, corrected.

The double assessments are guideline-compliant and adhere to a standardised and structured procedure, including reaching a consensus in the event of deviating findings.

## Methods / study design

### Quantitative research elements

The quantitative work packages of EVALUNG comprise core and exploratory components. They are all observational and based on administrative and medical routine data. These data are generated as part of the administration of all eligible persons and the management of the participants. No data is collected solely for the research project. The initial project stage involves analysing the documentation content and data structures to determine their availability, completeness, and suitability for the planned analyses.

The aim of the core components is to conduct cross-sectional analyses of participation and clinical outcomes. The aim of the exploratory components is to assess the feasibility of longitudinal analyses and record linkage with selected cancer registries. In addition, a comprehensive quality management concept is part of the quantitative elements, including the development and consensus of quality indicators.

### Legal basis for data processing and data protection concept

The legal basis for statutory accident insurance is found in the German Social Code Book (SGB) VII. Research into work-related health hazards and preventive measures is one of the main tasks of the statutory accident insurance. Within the EVA-LCS, the institutions naturally have to work with data that can identify individuals, such as name, address and date of birth. If the accident insurance institutions do not conduct the research themselves, they may only transmit the data in anonymised form to external bodies (§ 206 SGB VII, paragraph 5, sentence 1). Therefore, it is essential to develop a comprehensive data protection and management plan that is coordinated with the primary data owners.

### Study population

EVALUNG has been designed to evaluate the EVA-LCS as a whole. The generation of unbiased and reliable results is best achieved through a complete survey. This includes all potential participants of EVA-LCS, i.e. participants and non-participants. For example, established quality indicators such as participation rates or tumour detection rates can only be reliably reported on the basis of the entire screening cohort. As in the evaluation of organised cancer screening programmes (such as the German mammography screening programme), non-participants should be included in the evaluation as a relevant comparison group. Within the EVA-LCS, certain outcome parameters are also available from non-participants. This is because suspected lung cancer always triggers an OD investigation for OD no. 4104 in the EVA-LCS cohort. It does not matter where the suspicion first arose.

In summary, EVALUNG is an observational study with a complete survey of all persons who meet the inclusion criteria mentioned above (Table 1). As of December 2021, approximately 20,900 of all insured persons registered with the GVS meet the inclusion criteria for the EVA-LCS, i.e. they have been contacted at least once. In addition, there are approximately 1,300 individuals with recognised OD no. 4103 in the UVT cohort. The participation rate for the LDCT ranged between 30 and 40% per screening round, resulting in an estimated 6,500 to 8,500 cases with a scan. For the longitudinal analysis, data from 1,000 insured individuals over at least two screening rounds (initial and first follow-up) are expected.

### Selection of variables

The selection of variables from the EVA-LCS for the EVALUNG research project is theory-driven based on the dataset descriptions, taking into account the research questions, clinical endpoints and planned quality indicators, as well as data protection considerations (Table 2).

**Table 2:** Description of selected administrative and medical routine data from the lung cancer screening by the German Social Accident Insurance (EVA-LCS) that will be used for the evaluation

Categories	Variable
Demographic data of eligible persons	Anonymised identifier
	Year of birth
	Month of death (if applicable)
	Sex
	German federal state
	Occupational group (coded)
	Specific job function (coded)
	Name of accident insurance institution
Eligibility for EVA-LCS	Tobacco exposure (pack years)
	Smoke-quit (if applicable)
	Asbestos dust exposure (duration)
	Recognised OD “asbestosis” present
Invitation management and participation	Screening round
	Due date of next medical concultation
	Mailing date of invitation letter
	Mailing date of reminder
	Consent to participate in EVA-LCS
	Reason for non-participation
	Date of referral to occupational medicine
Medical consultation	Date of consultation
	Anamnesis of lung diseases
	Information on smoking cessation programmes
	Decision to have a scan
CT scan	Date of scan
	Body mass index (category)
	Radiation dose (millisievert)
	Radiological institute (anonymised)
	CT findings
	Control scan due (if applicable)
	Date of control scan
EVA-LCS follow-up	Next invitation due
	Withdrawal from EVA-LCS
	Reason for withdrawal
Occupational diseases record	Code of suspected OD
	Date of handover of case to responsible UVT
	Result of OD investigation
	Death suspected to OD
Tumour (lung cancer) details	ICD code
	TNM classification
	Grading
	False-positive finding

OD occupational disease, UVT German Social Accident Insurance Institution

### Data management

In preparation for data analysis, only the variables selected for EVALUNG according to the study protocol are extracted from the GVS databases. Prior to transmission, the data are anonymised. Possible identification variables, such as date of birth or postal code, will be coarsened. A person from the cohort is represented in the research dataset by an anonymised control number. As the allocation key is destroyed before the data is transmitted to the evaluation centre, this control number cannot be re-identified. As EVA-LCS is an ongoing screening programme, annual data exports and analyses are planned during the course of the EVALUNG project.

Although some basic statistics are available from the GVS, the quality of the individual data items remains to be ascertained. Given the nature of secondary use data, it is expected that a certain proportion of unexpected missing data will occur. Consequently, a missing data analysis will be conducted. In the event of anomalies, a discussion will be initiated with the primary data holder (GVS) to investigate the reasons for the missing data, such as technical issues (e.g. database export errors) or human factors (e.g. miscoding), and possible solutions. In instances of clinical issues, the relevant medical expertise will be requested.

The specific analysis and frequency will determine whether missing values are treated as separate categories or excluded from the analyses. The quality of the data and the specific handling of missing data will be addressed in the methods section of subsequent project-related publications.

### Data analysis

The following section describes the planned analyses, which are dependent on the availability of routine data. Descriptive methods and survival analyses (Cox regression analyses) will be used as statistical procedures.

#### Cross-sectional analyses

- Description of participation and participation patterns of eligible persons in the EVA-LCS according to age, gender, screening round, region and other characteristics.
- Selected outcome measures for evaluating the effectiveness of EVA-LCS, such as.
  - Detection rate of lung cancer in initial and follow-up screening rounds.
  - Tumour stages and other characteristics of lung cancer detected in EVA-LCS.
- Epidemiological test characteristics of LDCT screening (positive and negative predictive value, sensitivity, specificity, rate of false-positive findings).
- Rate of control scans within shortened interval.
- Rate of findings that require immediate further diagnostics.
- Rate of notifications and recognitions of ODs from EVA-LCS.
- Differences between the GVS cohort and UVT cohort with regard to the aforementioned outcome measures including detection rate and tumour characteristics.

#### Longitudinal analyses

These components of EVALUNG comprise preparation for longitudinal analyses using a sample of eligible individuals in a cohort study. These analyses are to assess feasibility and include a record linkage with data from selected cancer registries (further details below). The following outcome parameters will be analysed:

- Drop-out rate,
- Incidence of lung cancer and other cancers,
- Proportion of interval carcinoma,
- Rate of false-negative findings based on the observed interval carcinoma,
- Association of certain characteristics such as asbestos fibre years or pack years with the observed outcomes for detailed analysis of a dose-response relationship of the risk factors,
- Tobacco smoking and, if applicable, changes in smoking behaviour,
- Changes in the rate of advanced tumours (as a surrogate for the expected lung cancer mortality rate),
- Development of lung cancer-specific and all-cause mortality (if possible).

Additionally, EVALUNG will investigate the feasibility of making meaningful comparisons between the incidence and mortality rates of participants and non-participants. The study will also explore whether a specific subgroup of non-participants is suitable as a comparison group. This is important because participants and non-participants in screening programmes tend to differ in terms of health, socio-demographic, and socio-economic characteristics, which can lead to confounding. For this purpose, it is important to determine whether the necessary information is available and usable. In addition to age and the documented reasons for non-participation as recorded by the GVS, smoking behaviour, comorbidities and symptoms are variables for which the analysis can be adjusted for.

### Outcome measurements

The clinically relevant outcomes summarised below are derived from the core and exploratory components.

#### Primary outcome measures

- Participation rate in each screening round (incl. control CT-scans, if applicable),
- Detection rate of lung cancer in the annual screening rounds (incl. control CT-scans, if applicable),
- Tumour stages and characteristics,
- Notification and recognition of asbestos-related ODs in the annual screening rounds (incl. control CT-scans, if applicable).

#### Secondary outcome measures

- False-positive findings.
- Interval carcinoma (false-negative findings).
- Complication rate during (invasive) diagnostic evaluation of suspicious findings.
- Incidence of other cancers (except lung cancer).
- Mortality (lung cancer mortality, all-cause mortality, cancer mortality other than lung cancer).

### Record linkage with selected cancer registries

As an exploratory component, a record-linkage with selected cancer registries^4^ will be designed. The aim of this record-linkage is to complete and validate tumour details, record cancer incidence in non-participants, detect interval carcinomas and track vital status over time.

Pseudonymous data linkage without informed consent is possible in principle, but is limited to certain cancer registries [26]. From a German social legislation perspective, the use of non-anonymised data must be authorised by the Federal Office for Social Security as the supervisory authority (see § 75 SGB X). Approval for the use of pseudonymous data without informed consent is an exception, that requires appropriate justification and extensive technical and organizational measures to ensure adequate data protection. From a technical point of view, procedures based on encrypted identifiers can be used for this purpose [27]. Finally, it must be ensured that EVALUNG only receives anonymised data and that no individual can be identified.

### Development and piloting of quality indicators

In addition to evaluating outcomes, the EVALUNG research project aims to develop and implement a structured, integrated quality management system. In addition to outcome quality, it is also important to consider structural and process quality as well. To achieve this goal, EVALUNG aims to develop a suitable set of indicators for the EVA-LCS.

The methodology can be based on the development process of quality indicators in the German Guideline Program in Oncology [28], which can be summarised as follows:

- Composition of a representative and interprofessional panel,
- Search for and review of existing indicators, or the development of new indicators based on recommendations, taking into account available data,
- Standardised formal assessment of proposed and potential indicators in terms of.
  - Relevance,
  - Scientific soundness,
  - Practicability,
- Consensus,
- Pilot testing and validation.

The requirements for individual indicators should follow the RUMBA rule, which means that they must be *r*elevant, *u*nderstandable, *m*easurable, *b*ehaviourable (changeable through behaviour), and *a*chievable [29].

### Qualitative research elements

The EVALUNG project also includes conceptual and qualitative components. These include the development of a decision aid for the EVA-LCS participants and qualitative interviews on the satisfaction and perception of the EVA-LCS.

### Developing and testing of a decision aid

One of the qualitative aspects of EVALUNG is the development of an evidence-based decision aid for participants of EVA-LCS. Evidence-based decision aids aim to inform individuals about medical examinations or other procedures in a transparent and comprehensible manner, with a focus on presenting a balanced and neutral depiction of the potential harms and benefits of the procedure.

The currently available information on LCS for insured persons mentions the benefits of LDCT and also the radiation risk. However, it does not take all possible harms into account and quantify possible outcomes, such as the number of findings requiring further diagnostics, false-positive findings and the potential risk reduction from screening. Such figures are nowadays considered essential for evidence-based decision making [30, 31]. The decision aid is considered to be an independent part of EVALUNG, that has already been completed. However, for the sake of completeness, the methods used in developing and piloting it will be briefly outlined here.

A number of literature reviews were carried out to perform the development of the decision aid, including existing decision aids for LCS or other cancer screening programmes and methodologies. A first draft was developed, discussed and adjusted in cooperation with the affected parties. The revised draft was then piloted with EVA-LCS participants to ensure that the information is understandable and addresses the persons in an appropriate way. For the pilot testing, EVA-LCS participants of the Institute for Occupational and Maritime Medicine (ZfAM) were given the draft of the decision aid to read it at home before getting interviewed. The semi-structured interviews, which focused on the comprehensibility, information content and perceived usefulness of the decision aid, took place at the ZfAM and were audio-recorded. Finally, transcription and structured qualitative content analysis were conducted, resulting in further adjustments based on the testing results [32].

### Qualitative interviews with participants and physicians

One further aspect of the evaluation of the EVA-LCS is to conduct qualitative interviews with (a) persons from both cohorts, who undergo EVA-LCS (participants) and (b) participating physicians from the fields of occupational medicine, pulmonology and radiology (physicians). The aim of the interviews is to gain insight into attitudes towards the programme and experiences so far from both perspectives. This will help to identify potential problems and provide an opportunity to adjust the screening process and to ensure the quality of the programme.

For the participants, there are two main aspects of the qualitative evaluation of the EVA-LCS: (1) Perception and satisfaction with the screening and (2) Psychological effects experienced during participation, including anxiety while awaiting medical results and fear of cancer, as well as the impact on quality of life. The recruitment of the participants will be assisted by the GVS and the UVT, as they can provide the study information and invitation to the participants after they have agreed to participate in the EVA-LCS. This is also the inclusion criteria for the qualitative interviews. If the participants wish to take part in the qualitative study, they share their contact information with the research team by responding to the study invitation. Around 200 persons from the GVS-cohort and further 100 persons from the UVT-cohort will be contacted, with an attempt to achieve a balanced regional, gender and age distribution. Participants will be offered an incentive to take part in the study.

The persons will undergo five interviews throughout the study, starting after the initial invitation to the EVA-LCS and ending at a 3-month follow-up after receiving the LDCT results. The accompanying (“real time”) evaluation can reduce recall bias and allows to get an insight into the perception of the current process as well as the psychological effects at each specific time point in the programme (Table 3).

**Table 3:** Schedule and contents of the qualitative interviews with participants of lung cancer screening offered by the German Social Accident Insurance (EVA-LCS)

Contents	Experience with EVA-LCS in relation to…					Psychological effects
**Time schedule**	Perception & expectations of LDCT	Invitation procedure	Appointment procedure & medical consultation	Procedure of LDCT scan	Communication of the findings	Anxiety, Quality of life, etc.
**After receiving the invitation** **(initial interview, including study administration)**	✓	✓				✓
**After medical consultation** **(first follow-up)**	✓		✓			✓
**Directly after the LDCT scan** **(second follow-up)**				✓		✓
**After communication of the findings** **(third follow-up)**	✓				✓	✓
**3 months after communication of the findings** **(final interview)**	✓					✓

The interviews with the participating physicians will focus on their attitudes and knowledge regarding the EVA-LCS. These physicians will be recruited by the research team. It is planned to contact those occupational physicians and pulmonologists with at least 20 EVA-LCS consultations per year, which currently applies to 98 physicians. Furthermore, the 35 radiologists or radiological institutes with the highest number of EVA-LCS cases will also be contacted in order to achieve a balanced allocation of all three professions involved.

For each of the two groups, i.e. the participants and the physicians, 25 interviews will be sought, either in person, by telephone or via video call. All interviews will be transcribed and analysed using structured qualitative content analysis. Based on the results of the qualitative interviews, a questionnaire will be prepared to enable regular surveys in the future.

## Discussion

The EVALUNG health care research project aims to develop and implement a scientific evaluation concept for the EVA-LCS combining quantitative and qualitative approaches. The quantitative analyses will be based solely on anonymised routine administrative and medical data. The additional use of qualitative research elements may identify further areas for improvement of the EVA-LCS. These include, for example, the perception and satisfaction of the participants as well as attitude and knowledge of the physicians involved.

### Strengths and limitations

One of the strengths of the EVA-LCS cohort is its clear definition. Due to former occupational asbestos exposure, there is a legal obligation for GVS and UVT to provide medical follow-up care. Outcome parameters relevant to the EVA-LCS, such as lung cancer and related occupational diseases, are available not only for participants but for the entire eligible cohort. Regular vital status checks are conducted, and if necessary, death certificates are requested to determine the cause of death.

As with all secondary analyses, only information documented in a structured format can be used for evaluation. Data quality determines the usability of existing data sets for EVALUNG. An assessment of data quality will be conducted after transmission and verification of the real data. For this reason, some of the planned analyses may be limited.

Although documentation is predominantly digital using a standardised web-based platform, data gaps and breaks in documentation may occur. This may be the case when an eligible person changes from the GVS cohort to the UVT cohort or when the statutory health insurance becomes responsible for treatment instead of the statutory accident insurance. Whether such a change is associated with loss to follow-up will only become clear in combined longitudinal analyses of both cohorts.

The evaluation of the EVA-LCS outcome parameters occurs on two distinct levels. Accurate *clinical information*, including tumour details such as TNM staging, is essential for medical assessments to identify any potential shift towards more favourable prognoses in tumours. On the other hand, the *result of an OD investigation* is important to the statutory accident insurance from its administrative perspective. Early detection and recognition of an OD can be advantageous for those affected, regardless of further tumour details.

Linkage with cancer registry data is an option to compensate for these limitations. It will be explored to complement and validate information on tumour characteristics, and to assess interval carcinoma as well as lung cancer in non-participants.

### Outlook

Currently, the statutory health insurance in Germany is considering the introduction of a general lung cancer screening programme with LDCT for high-risk groups based on smoking history and age [33]. Although the EVA-LCS is not subject to the regulations for organised cancer screening programmes in Germany, it meets many of the structural requirements stipulated therein. The EVA-LCS consists of an invitation procedure coordinated by the accident insurance institutions, a medical consultation to support informed decision making, and LDCT scans according to a standardized protocol in quality-assured radiological facilities. Therefore, EVALUNG will not only evaluate the EVA-LCS, but may also provide valuable experience for the planning and development of a national LDCT lung cancer screening programme in Germany [33].

## Data Availability

In accordance with data protection regulations, the quantitative data have been provided on the condition that they will not be passed on to third parties. The datasets generated and analysed for EVALUNG are therefore not publicly available. The qualitative data are also not publicly available, as they may contain information that could compromise the privacy of the research participants.

## Footnotes

1 Statutory accident insurance is an arm of the social insurance system in Germany. As a mandatory insurance scheme, it provides compensation for damage to health suffered by insured persons in the course of their insured work activities. Therefore, its aim is to prevent not only accidents but also other work-related illnesses. (Source: https://www.bmas.de/EN/Social-Affairs/Statutory-accident-insurance/statutory-accident-insurance.html)

2 Participating accident insurance institutions: German Social Accident Insurance Institution (Berufsgenossenschaft – BG) for the building trade (BG BAU), BG for the energy, textile, electrical and media products sectors (BG ETEM), BG for the woodworking and metalworking industries (BGHM), BG for the trade and logistics industry (BGHW), BG for the raw materials and chemical industry (BG RCI), BG for the administrative sector (VBG).

3 Legal definition for OD no. 4104: Lung, larynx or ovarian cancer combined with asbestosis, combined with diseases of the pleura caused by asbestos dust or if there is evidence of cumulative exposure to asbestos dust in the workplace of at least 25 fibre years (25 × 10^6^ [(fibre/m³) x years]).

4 In Germany, almost every federal state has its own cancer registry and its own cancer registry act.
